# Compact Laser-Induced Fluorescence Detector with Adjustable Laser Focal Spot for Multiple Purposes

**DOI:** 10.3390/s24196224

**Published:** 2024-09-26

**Authors:** Zihe Xu, Xi Chen, Fangwu Liu

**Affiliations:** 1 Shanghai Institute of Technical Physics, Chinese Academy of Sciences, Shanghai 200083, China; xuzihe@mail.sitp.ac.cn; 2 University of Chinese Academy of Sciences, Beijing 100049, China; 3 Shanghai Key Laboratory of Atmospheric Particle Pollution Prevention (LAP3), Department of Environmental Science and Engineering, Fudan University, Shanghai 200433, China; 20110740003@fudan.edu.cn

**Keywords:** laser-induced fluorescence, epifluorescence, adjustable, handheld, limit of detection

## Abstract

In many research fields, the demand for miniaturized laser-induced fluorescence (LIF) detection systems has been increasing. This work has developed a compact LIF detector, employing a laser diode as the excitation source and a photodiode as the photodetector with an adjustable laser focal spot, to meet the diverse requirements of various observation targets, such as capillaries, PCR tubes, and microfluidic chips. It features the functionalities of background fluorescence correction, the adaptive adjustment of the dynamic range, and constant power control for the laser. The influence of the excitation power on the detection limit was studied through experiments, and the configuration results for LED/LD as light sources and 487/450 nm wavelengths were compared and optimized. A fully integrated, compact, modular epifluorescence LIF detector was subsequently constructed, measuring 40 × 22 × 38 mm^3^ in total size, with a cost of USD 320, and achieving a detection limit of 0.4 nM for fluorescein sodium. Finally, the detector was integrated into a nucleic acid detection system with a microfluidic chip on the Chinese Space Station (CSS) and was also tested with PCR tubes and capillaries, proving its broad practicality and adaptability to various analytical systems.

## 1. Introduction

Laser-induced fluorescence (LIF) is a highly sensitive and specific detection technique, widely used in capillary electrophoresis (CE) [1,2,3], flow cytometry [4,5], and high-performance liquid chromatography (HPLC) [6,7]. In practical applications such as point-of-care testing (POCT) [8,9], biomedicine [10,11], and chemical analysis [12], there has been a growing demand for LIF detection modules to be miniaturized, modularized, and simplified, thereby expanding their application scope significantly.

The optical configurations of LIF detectors, as well as the choices of excitation sources and detectors, vary widely. Weaver et al. developed a confocal laser-induced fluorescence detector for sub-micron capillary systems, utilizing a laser diode (LD) and a photomultiplier tube (PMT). In a 2-μm-i.d. capillary flow injection analysis system, they achieved a detection limit as low as 14 zeptomoles for fluorescein [13]. Fang et al. designed a compact LIF detector adopting a quasi-confocal optical configuration, integrating a 450 nm LD and a miniaturized PMT as well as other optical and electronic modules. The total dimensions were 9.1 × 6.2 × 4.1 cm^3^, with an overall cost of about USD 2000, achieving a detection limit of 0.42 nM for a fluorescein solution [14]. Geng et al. developed a compact and low-cost LIF detector for capillary electroosmotic driven systems, using a 450 nm LD and a proprietary PD (AccuOpt2000), reaching a detection limit of 3 pM for sodium fluorescein [15]. Pan et al. developed a miniature handheld high-speed capillary system integrated with an orthogonal LIF detection module. The fluorescence was collected in a plane perpendicular to the laser beam at a 45° angle to the capillary. The device measured only 90 × 75 × 77 mm^3^, reducing the instrument cost to approximately USD 500, with a detection limit of 1.02 nM for a sodium fluorescein solution [16]. Peng et al. constructed miniaturized LIF detection systems with three different optical configurations, investigating the detection performance when the excitation light and photodetector were in orthogonal, confocal, and oblique positions, respectively. They optimized the filtering and focusing methods, eventually utilizing an orthogonal configuration, with total dimensions of 50 × 20 × 46 mm^3^ and a total cost of USD 380, achieving a detection limit for a fluorescein solution of 10 pM [17]. Oborny et al. developed an LIF detection system suitable for high-radiation environments for the Europa landing mission, employing LDs composed of high-radiation-tolerant gallium nitride and a miniaturized PMT. It could detect amino acids at 1 nM under extreme radiation conditions of 300 krad [18].

In addition, commercial detectors such as the SCIEX PA 800 Plus Pharmaceutical Analysis System are equipped with an LIF detector that utilizes a solid-state laser with an excitation wavelength of 488 nm. It employs dual light sources at a 45-degree angle to illuminate the target and uses a ball lens to collect fluorescence [19]. However, commercial detectors have the disadvantage of being bulky and expensive. Other commercial fluorescence detection modules, such as the MONAISI MMFD-470 and DICP mFLD, often use LEDs as the excitation source to reduce the size and cost, yet they are unfit for capillary systems.

In most research and commercial equipment, LIF detectors are used mainly for capillary systems and rarely for large liquid volume devices. In this work, we constructed a miniaturized fluorescence detection module based on an epifluorescence optical configuration, which can be applied to both fields in analytical chemistry and POCT. We used a 487 nm semiconductor LD as the excitation source and a photodiode (PD) as the photoelectric detector. The module is equipped with functions such as constant power control for the excitation source, the adjustment of the laser focus spot, background correction for the fluorescence detector, and an adjustable dynamic range. We studied the effects of various factors on the detection performance, including the filter system, choice of excitation device, and excitation wavelength. Based on the optimization results, we fully integrated the optical module, detector, laser, and main control circuit, ultimately constructing a handheld LIF detection system with dimensions of only 40 × 22 × 38 mm^3^ (including electronic components), for USD 320. This module is smaller, cheaper, and maintains the performance of those previously reported LIF systems constructed with PD detectors. This module can work independently and is flexibly adaptable to various analytical systems. We have implanted this module into the Microbial Online Monitoring Module (MOMM) for the CSS and tested it with PCR tubes and capillaries, eventually achieving similar LODs for various targets, as low as 0.4 nM.

## 2. Materials and Methods

In this section, we will state all of the experiment materials that we use, including the fluorescein solution, LAMP assay, and the components of the LIF system. Then, we will elaborate on the experimental conditions and procedure.

### 2.1. Fluorescein Solution for LOD Analysis

The LOD analysis was performed using a 10 mM solution of fluorescein sodium. This was prepared by dissolving 3.76 mg of fluorescein sodium salt (IF1420, Solarbio, Beijing, China) in 1 mL of 5 mM sodium tetraborate buffer standard solution (BYS6000, Solarbio, Beijing, China, pH 9.18). The stock solution was subsequently diluted with sodium tetraborate buffer to prepare a series of gradient fluorescein solutions. The concentrations of the series solutions were 0 nM, 1 nM, 5 nM, 10 nM, 15 nM, 25 nM, 50 nM, 100 nM. All chemicals used were of analytical grade and deionized water was used throughout.

### 2.2. LAMP Assay

LAMP reaction solution preparation and lyophilization: 2× Indicator LAMP Master Mix with Dye (for-Lyo) 12.5 μL (FLE225, Novoprotein Scientific Inc., Suzhou, China), 100 mM MgSO_4_ 1.5 μL (Sigma Aldrich Merck KGaA, Darmstadt, Germany), 10 mM dNTP 3.5 μL (E110, Novoprotein Scientific Inc., Suzhou, China), and 1 μL primer set mix solution were used to configure the sampling solution. Six primers were included in the primer set mix, comprising two inner primers (FIP, BIP), two outer primers (F3, B3), and two loop primers (LF, LB). The primers were designed using the Primer Explorer V5 design tool (https://primerexplorer.jp/lampv5/index.html, accessed on 2 February 2021), and their design sequences are shown in Table S1. The specificity of the primer set and target sequence was analyzed using the BLAST tool (http://www.ncbi.nlm.nih.gov/, accessed on 2 February 2021). The primers were synthesized by BiOligo Biotechnology (Shanghai, China) Co., Ltd. The primers were mixed in DEPC water. The final concentration of FIP and BIP was 1.6 µmol/L, that of F3 and B3 was 0.2 µmol/L, and that of LF and LB was 0.8 µmol/L. Bst DNA polymerase lyophilized beads (Lyobead Bio Tech Inc., Suzhou, China), working as *Taq* in the PCR, were preset in the amplification chamber. DNA was extracted from standard bacterial strains by a commercialized DNA extraction kit (QIAamp DNA Mini Kit, Qiagen, Hilden, Germany). The concentration of the E. coli DNA template suspension was 5 × 10^6^ copies∙μL^−1^. The mixture was incubated at 63 °C for 30 min. The negative control (NC) group was the no-template control.

### 2.3. Building of the LIF Detection System

**Figure 1:**
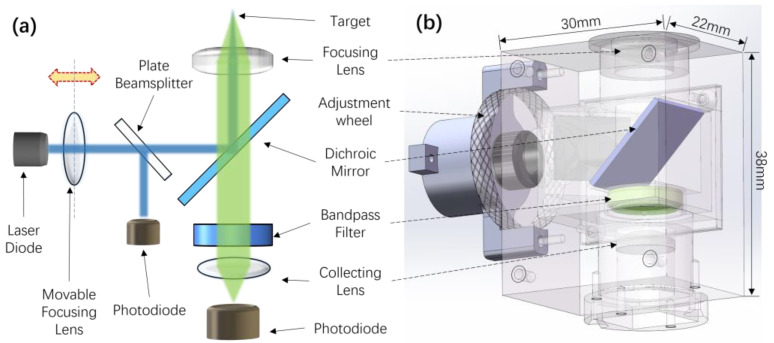
(**a**) Schematic diagram of the optical configuration of the LIF detection system. The focusing lens of the LD can move along the optical axis, as indicated by the arrow. (**b**) Structural schematic of the LIF detector.

The optical configuration of the epifluorescence LIF detection system is shown in Figure 1a and the structure is shown in Figure 1b. A 450 nm or 487 nm LD (GH04580A2G/GH04850B2G, SHARP) is used as an excitation source. The laser beam first passes through a converging lens group (HL0910.5, BLASER, EFL 6 mm) and then reaches a 45° flat plate beamsplitter (#46-664, Edmund Optics, 25/75 Standard Beamsplitter). Then, 25% of the laser energy is directed towards a PD (S13373, Hamamatsu Photonics) to monitor the laser intensity, while the majority reaches a dichroic mirror (#34-732, Edmund Optics, HP Ultra Dichroic 495 nm). This dichroic mirror exhibits high reflectance in the 435–488 nm range and high transmittance above 495 nm. After reflection, the excitation laser is focused onto the target via a plano-convex lens (#63-484, Edmund Optics, d10 × f10). The emitted fluorescence is collected by the same lens, passes back through the same dichroic mirror, and is then filtered by a 540 nm bandpass filter (#86-344, Edmund Optics, FWHM 56.00 nm). It is subsequently focused onto the detection window of a PD (S1226-44BK, Hamamatsu Photonics) by another plano-convex lens (#45-082, Edmund Optics, d9 × f9). The laser converging lens is adjustable along the optical axis to calibrate the laser beam and focal spot size. By adjusting the excitation focus spot without changing the distance, differently sized and shaped targets can be observed, such as droplets in capillary tubes, cylindrical reaction wells on microfluidic chips, or PCR tubes. All components are fixed in a 3D-printed case.

### 2.4. Positioning of the Detection System to the Target

The detection module is secured, and the target is positioned on a translation stage 10 cm away from the detection lens. First, the position of the laser focusing lens is adjusted until the smallest and brightest laser spot is observed on the target, and the position of the laser focusing lens is locked. Then, the position of the target on the translation stage is slightly adjusted to ensure that it is aligned with the optical axis. After obtaining the maximum fluorescence signal, the position of the target is locked. When the detecting target is changed, we turn the adjustment wheel anticlockwise to defocus the laser until the excitation beam covers all areas of the detecting target.

### 2.5. Observation Process

A series of gradient concentration fluorescein solutions is prepared and used immediately. Then, they are tested in order from low to high concentration. Each concentration is tested 20 times; then, we derive the average and standard error. All LOD tests follow this procedure.

For LAMP reagents, a timed collection method is used. During the amplification process, a fluorescence intensity signal is collected every 30 s. Once the process is complete, an amplification fluorescence curve can be obtained.

## 3. Construction of the Electronics System

**Figure 2:**
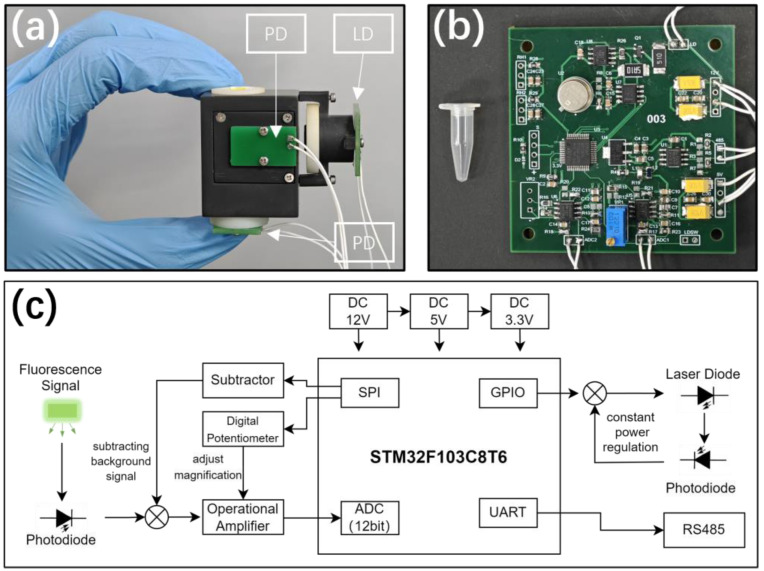
(**a**,**b**) Images of the LIF detector and PCB board. (**c**) Circuit schematic of the PCB board.

Images of the PCB board and the circuit schematic are shown in Figure 2b,c and Figure S2. The MCU utilizes an STM32F103C8T6, a 32-bit ARM microcontroller, which integrates various peripheral functions, including ADC, SPI, DMA, and UART. The external power input is 12 V DC, which is converted to 5 V and 3.3 V, supplied through onboard LDO chips. The UART of the MCU handles communication data and converts the signal to the RS485 format via a conversion chip.

### 3.1. Adjustment of Dynamic Range and Background Correction

The fluorescence collection pathway consists of one signal collection channel and two feedback loops. The PD converts the received fluorescence signal into a photocurrent, which is then transformed into a voltage signal via a sampling resistor, and then into an appropriately scaled voltage signal that is fed into the 12-bit ADC of the MCU through a series of op-amp circuits. One feedback loop involves the MCU controlling a digital potentiometer to adjust the gain of the operational amplifier. A good fluorescence detection module should have a wide linear dynamic range, meaning that it can handle fluorescence intensities spanning from very low to very high levels without saturation or distortion [20,21]. Thus, we designed an adjustable gain feature to ensure the complete collection of the fluorescence signal. The other feedback loop consists of the MCU controlling a subtraction circuit to deduct the background signal acquired by the detector for correction, which can improve the sensitivity.

### 3.2. Constant Power Control of the Laser Source

We implemented a feedback control loop to stabilize the optical power output. The MCU controls the bias voltage of the transistor to modulate the driving current through the LD, which controls the output power. A portion of the laser beam is directed via a beamsplitter onto a PD. The resulting photocurrent from the PD is digitized via analog-to-digital conversion (ADC), allowing real-time optical power monitoring. The MCU processes the AD measurements and adjusts the control voltage accordingly to maintain a stable LD output at a setpoint, thereby achieving laser power stabilization.

## 4. Results and Discussion

### 4.1. Optimization of Optical Components

Epifluorescence configuration is widely used in LIF systems. To achieve miniaturization, short-focal-length lenses (9 and 10 mm) were implemented. However, it is possible that placing the PD for laser power control behind the dichroic mirror, instead of using a beamsplitter, could reduce the weight, cost, and optical complexity. We will investigate this promising idea in later research due to the limitations of this work.

**Figure 3:**
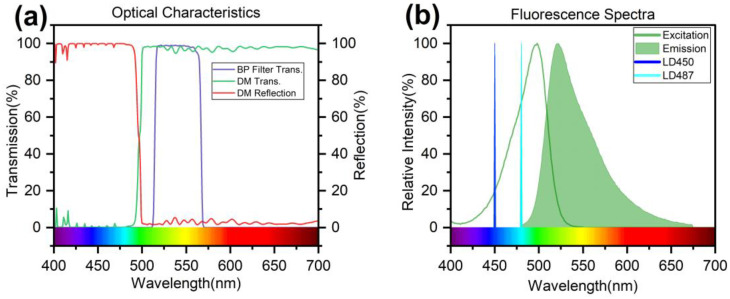
(**a**) Emission spectrum of laser and fluorescence spectrum of fluorescent dye. (**b**) Reflectance and transmittance of the dichroic mirror and bandpass filter used.

We chose suitable filters for this epifluorescence system. For SYBR Green I, with peak absorption at 497 nm and emission at 520 nm (Figure 3b), the dichroic mirror and filters must avoid crosstalk [22]. As shown in Figure 3a, using a spectrometer (HR4000, Ocean Insight), the dichroic mirror reflects over 98% at 491 nm and transmits over 98% above 501 nm. The emission filter, a 540 nm bandpass filter with a full width at half maximum (FWHM) of 56 nm, transmits 57.87% of the emission energy. Typically, a pinhole is used in the system to eliminate scattered light; however, it requires a relatively long focusing distance and limits the ability to detect large targets. Therefore, we use a bandpass filter to compensate partly for the absence of a pinhole. This setup ensures that minimal excitation light reaches the PD, improving the signal-to-noise ratio (SNR).

### 4.2. Optimization of the Excitation Source

Typically, an LD has advantages over an LED, such as a higher energy density and superior monochromaticity, collimation, and coherence of the output light, enabling a higher SNR. Thus, LDs are more favored in LIF systems [23]. However, the disadvantages include higher heating and poorer thermal stability and lifetimes. Our solutions include enhancing heat dissipation and employing constant power control, where a PD monitors the LD output in real time. Closed-loop control maintains a stable and adjustable optical power output.

**Figure 4:**
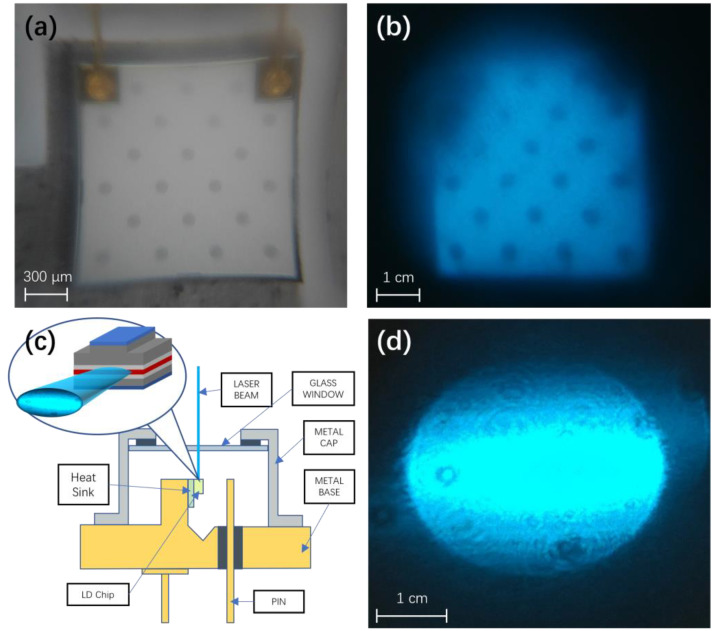
(**a**) Image of LED emitter element. (**b**) Emitted light pattern of LED. (**c**) Schematic diagram of LD structure. (**d**) Emitted light pattern of LD.

The light pattern of an LED is determined by the emitter geometry, commonly a rectangular profile at the mm scale, as shown in Figure 4a,b. The focused LED light spot is much larger than a laser and more suitable for targets like PCR tubes. A cross-section of the LD is shown in Figure 4c, where the red active layer measures at the μm scale. These edge emitters produce an output beam pattern as shown in Figure 4d. The tighter focusing achievable with LDs is apparent, with the light beam at the micron scale, appropriate for small targets in capillaries or microfluidic chips.

We measured the emission spectra for the 470 nm LED, 450 nm LD, and 487 nm LD, as shown in Figure S1. The LED has a 92 nm linewidth, while the lasers have monochromaticity with a 3 nm linewidth. Therefore, an excitation filter is needed for LEDs (blue line in Figure S1) but not for lasers, reducing the overall system cost.

### 4.3. Adaptive Adjustment of Dynamic Range

**Figure 5:**
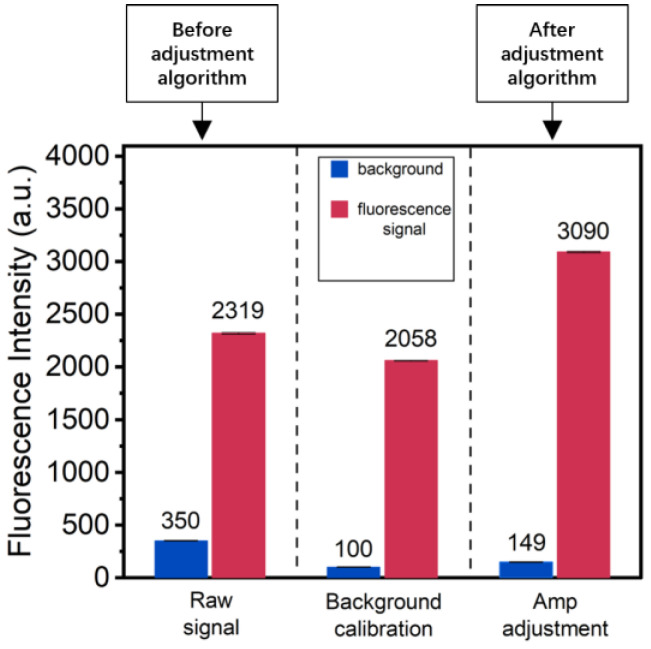
Testing of the module’s signal processing functions through three steps: First, before the adjustment algorithm begins, we collect the raw signal (**left**). Then, the background correction is performed (**middle**). Lastly, the amplification factor adjustment is performed, and we obtain the data after the adjustment algorithm (**right**). Fluorescence signals are measured 40 times in each step. The 487 nm LD is used for this test.

The module implements an adjustable dynamic range through three main steps: acquiring the raw signal, performing background correction, and adjusting the amplification factor. We tested this functionality, and the experimental results are shown in Figure 5. Initially, we collected the raw fluorescence signal and background signal. Background correction was then performed, subtracting 70% of the background and noise. Finally, the amplification factor was adjusted to amplify the maximum fluorescence signal to 75% of the ADC limit (the 12-bit ADC limit is 4095). The experiment demonstrated that this design effectively reduces background noise while appropriately amplifying the effective fluorescence signal, enhancing the effective signal by 1.5 times (amplification factor range from 6.1 to 1000). The signal-to-background ratio (SBR) also increased from 6.6 to 20.3 after the adjustment, aiding in the identification of weaker signals. This improvement could allow for the detection of a wide range of fluorescence concentrations.

### 4.4. Constant Power Control of the Light Source

**Figure 6:**
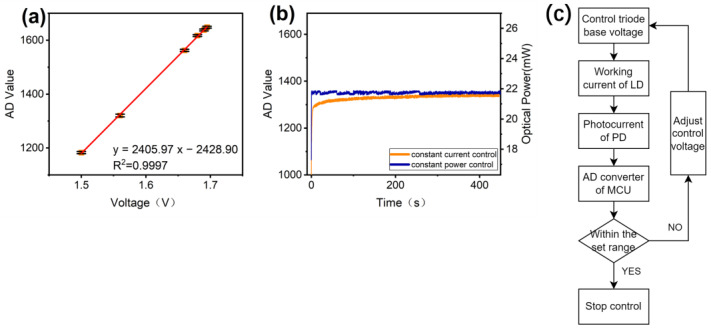
(**a**) Linear fit of control voltage versus feedback PD’s AD value for LD intensity. (**b**) Measurement of constant current control vs. constant power control for light source using an optical power meter. The LD is tested under the same room temperature and cooling conditions. (**c**) Flowchart of constant power control for the light source. The 487 nm LD is used for this test.

The intensity and stability of the excitation source directly impact the detection limits. With advancements in fabrication processes and control devices, the stability of LDs has improved significantly with adequate heat dissipation and incorporation control systems. However, commercially available TO-18 packaged LDs lack an integrated PD for feedback control. Thus, this work implements an additional feedback loop comprising a beamsplitter and PD to achieve constant power control. First, the linear relationship between the LD control voltage and feedback PD analog-to-digital (AD) value is established, as depicted in Figure 6a. The control voltage setting resolution is 0.001 V, while fluctuations up to ±10 AD counts are observed from the feedback PD. Applying dead zone control, an AD threshold value of 20 is set, which, from the linear fit, corresponds to a minimum control voltage step of 0.0083 V, rounded to 0.009 V. The control process is illustrated in Figure 6c.

By programming the above parameters and control logic into the MCU to run automatically, the constant power control of the excitation source can be realized. The output power was measured using an optical power meter (PM100D, Thorlabs, Newton, NJ, USA) aligned to the detection window, as depicted in Figure 6b. The orange line shows the constant current control results. Upon powering up, the optical output increases gradually, stabilizing after 300 s with a 0.92 mW maximum delta from the moment of powering on. The blue curve represents power-stabilized control. The optical power output is constant overall, with a few step changes corresponding to the feedback loop activating to adjust deviations. Ultimately, the power accuracy can achieve a maximum deviation of 0.36 mW from the set value, which is over twice as precise as constant current control. By stabilizing the excitation power, the detection is more reliable and accurate. Meanwhile, issues of output drift from heating or semiconductor aging over long-term operation are addressed. This capability is crucial for the long-time operation of analytical instruments, where the stability of the operating conditions and measurements is mandatory compared to simple constant current control.

### 4.5. LOD Using 450/487 nm Excitation Source

**Figure 7:**
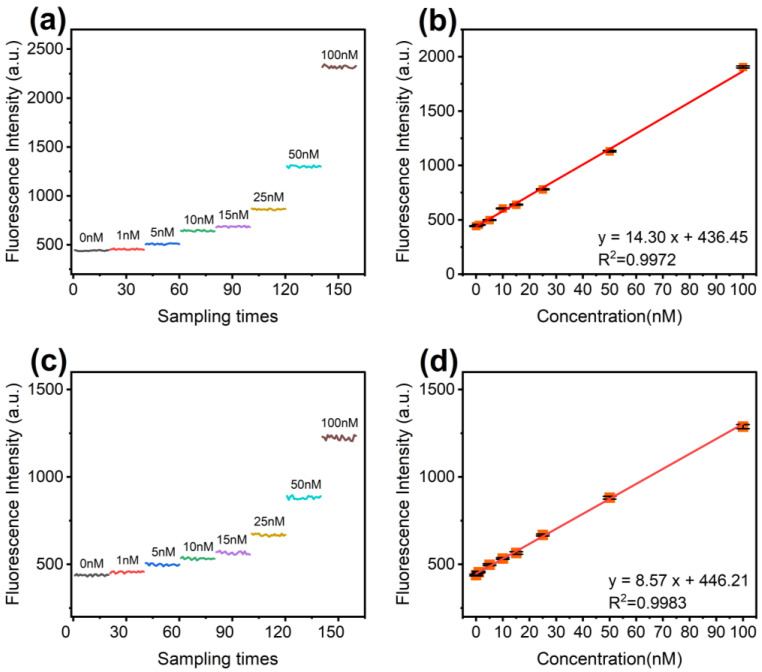
Fluorescence intensity recorded using 487 nm laser excitation (**a**) and the linear relationship between the fluorescence intensity and fluorescein concentration (**b**). Fluorescence intensity with 450 nm laser excitation (**c**) and corresponding linear fit (**d**).

Compared to expensive argon lasers and bulky peripherals, LDs offer a small footprint, low cost, and wavelength versatility, making them ideal for compact portable devices. The 450 nm LD is well established, offering performance stability at lower costs and widespread usage. The 487 nm LD, although better suited for fluorophore excitation, is a newer product with limited market validation and costs more than twice as much as the 450 nm LD. As shown in Figure 3a, the excitation efficiency for SYBR Green I is 20.07% at 450 nm compared to 85.87% at 487 nm, a significant increase. We evaluated the limit of detection (LOD) performance between these two excitation wavelengths. The LOD was defined at a signal-to-noise ratio (SNR) of 3, where noise is the standard deviation of 0 nM fluorescein. Here, 25 mW excitation power was used and gradient dilutions of fluorescein (25 µL) were prepared, and the fluorescence intensity was recorded across the concentrations, as shown in Figure 7. The data exhibited excellent linearity, with an R^2^ above 0.99. The derived LODs were 0.8 nM for 487 nm excitation and 1.5 nM for 450 nm excitation. While we expected approximately three to four times higher sensitivity with the 487 nm source, the noise was also higher, likely due to the 487 nm wavelength approaching the performance limit of the dichroic optics. Despite this, the significant gain in excitation efficiency translates to better detection limits, leading us to adopt the 487 nm excitation source.

### 4.6. Effect of the Adjustable Laser Focal Spot

**Figure 8:**
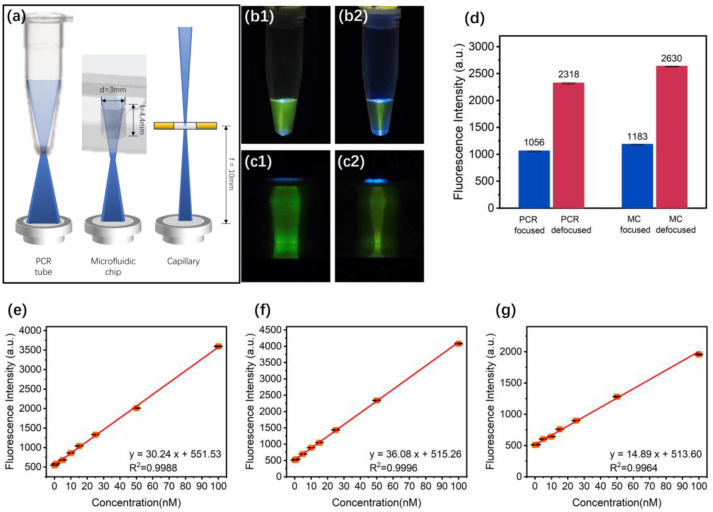
(**a**) Schematic diagram of adaptation of excitation beam for different detection targets and adapted beam size for PCR tubes (**b1**) and microfluidic chips (**c1**). Adjust beam size to minimum focused spot size (D = 80 μm) for capillary and then use on same PCR tubes (**b2**) and microfluidic chips (**c2**). Fluorescence intensities were collected under these four conditions (**d**), where PCR wide corresponds to (**b1**), PCR narrow to (**b2**), MC wide to (**c1**), and MC narrow to (**c2**). LOD for PCR tube (**e**), microfluidic chip (**f**), and capillary (**g**).

We implant the adjustable laser focal spot to enable this detection system to be adapted to various analytical systems. Since different detection targets require excitation light irradiation areas of various shapes and sizes, the excitation light should illuminate all areas containing fluorescent reagents while avoiding shining on the container because it generates background fluorescence, light scattering, and reflections. As shown in Figure 8a, the excitation of a PCR tube requires a conical beam, a microfluidic chip requires a slender cylindrical beam, and a capillary requires a tightly focused spot. An appropriately sized excitation beam ensures the maximum excitation efficiency and minimum fluorescence background, aiding in improved detection limits. Thus, an adjustable beam focusing module is implemented for the LD, as depicted in Figure 1b. By rotating the adjustment wheel, the relative distance between the coupling lens and LD can be varied, shaping the output beam. With the objective lens focal length kept constant, i.e., under the condition of unchanged fluorescence collection efficiency, the excitation beam is tailored to accommodate detection targets of various shapes.

The series of fluorescence solutions and the 25 mW 487 nm LD source were used. The diameter of the tip of the 0.2 mL PCR tube is 3.8 mm, the diameter of the chamber of the microfluidic chip is 3.5 mm, and the diameter of the capillary is 100 μm. The fluorescence signal of these targets is within the photodiode’s active area. The excitation time for each measurement is 0.3 s. The excitation beams were adapted for both PCR tubes and microfluidic chips, as shown in Figure 8(b1) and Figure 8(c1), and compared with the minimum focused spot size (D = 80 μm) achievable for capillaries, pictured in Figure 8(b2,c2). It is apparent that the adapted beams fully cover the reagent areas while avoiding most of the side walls of the containers. The fluorescence intensity in Figure 8d confirms that the adapted beam achieves similar detection limits (black line and blue line) for both targets, while an inappropriate laser focus spot results in over twice the fluorescence loss (red line and green line). Then, we test the LOD for the PCR tube, the chamber of the microfluidic chip, and the capillary, as shown in Figure 8e–g. The derived LODs are 0.4 nM, 0.5 nM, and 1.2 nM, respectively (SNR = 3), which meet most of the requirements for detection and analysis. This indicates that the adjustable excitation beam can be optimized for various targets to achieve a better LOD.

### 4.7. LAMP Fluorescence Amplification Testing

**Figure 9:**
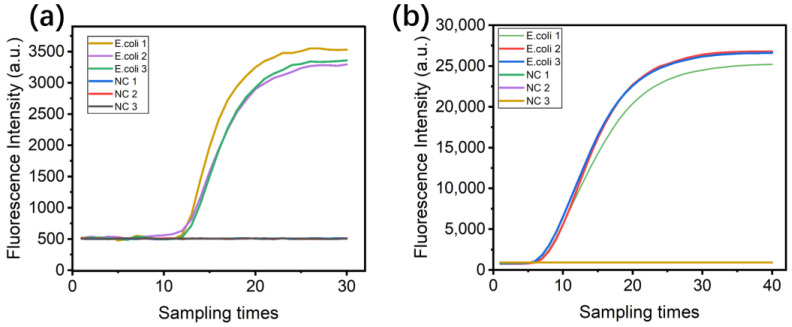
Real-time fluorescence curve of LAMP generated by this work (**a**) and a commercial qPCR system (**b**).

This detection system was used in the Microbial Online Monitoring Module aboard the CSS [24,25], integrating a centrifugal microfluidic chip (Figure S3) for LAMP-based nucleic acid amplification and fluorescent labeling to identify microbes. LAMP reagents were first combined as a 23 μL mix, and then 1 μL E. coli template and 1 μL polymerase were added; the combination was gently mixed and heated to 63 °C to initiate the amplification reaction. Three replicate tests were performed, each with positive and negative controls, collecting the fluorescence every 30 s to generate the kinetic amplification curves in Figure 9a. The same tests were carried out on a real-time quantitative PCR detection system (FQD-96A, BIOER, Hangzhou, China) and using the analysis software named Gene-9600 (Figure 9b). The results demonstrate the normal functioning of fluorescence detection with smooth curves and a stable readout. The consistency observed across the independent experiments indicates the high assay reproducibility and reliability.

## 5. Conclusions

In this work, we have developed a stand-alone, compact epifluorescence laser-induced fluorescence detection system using an LD for excitation and PDs for readout. Key attributes include background signal correction, an auto-scaling dynamic range, and excitation power stabilization. Further, the adjustable beam size maintains the performance when detecting various targets, enabling versatility. We optimized the excitation power, excitation source selection, and excitation wavelength to achieve a detection limit of 0.4 nM for fluorescein sodium. This detection system has also been compared with the previously reported compact LIF detectors (Table 1), showing its advantages in size and cost while maintaining comparable performance. Applicable to both nanoliter droplets and microliter chambers, this LIF detector has broad utility. Currently, this detector has been implemented in the Microbial Online Monitoring Module for the CSS and it can be integrated into diverse analytical platforms for a small footprint, independent capabilities, and adaptability.

## Figures and Tables

**Table 1:** Comparison of key features of different compact LIF detection systems.

Size	Cost (USD)	Optical Configuration	Excitation Source	Photodetector	Analyte	LOD	Ref.
9.1 × 6.2 × 4.1 cm^3^	2000	Quasi-confocal	450 nm LD	PMT	Fluorescein	0.42 nM	[14]
29 × 25 × 15.5 cm^3^	Over 500	Confocal	450 nm LD	PD (AccuOpt2000)	Fluorescein	3 pM	[15]
90 × 75 × 77 mm^3^	500	Orthogonal	450 nm LD	PD	Fluorescamine-labeled proteins	1 nM	[16]
50 × 20 × 46 mm^3^	380	Orthogonal	450 nm LD	PD	Fluorescein	10 pM	[17]
Not available	Not available	Confocal	405/488 nm LD	PMT	Amino acids	1 nM	[18]
40 × 22 × 38 mm^3^	320	Epifluorescence	487 nm LD	PD	Fluorescein	0.4 nM	This work

## Data Availability

Dataset available on request from the authors.

## Supplementary Materials

The following supporting information can be downloaded at: https://www.mdpi.com/article/10.3390/s24196224/s1, Figure S1: Spectrum of the light source; Figure S2: Circuit schematic of the control board; Figure S3: Image of the centrifugal microfluidic chip. Table S1: Primer set sequencing and their position in genomes.
