# Supplementary material for: Compact Laser-Induced Fluorescence Detector with Adjustable Laser Focal Spot for Multiple Purposes
Source: Sensors (Basel). 2024 Sep 26;24(19):6224. doi: 10.3390/s24196224 (PMC11479135; doi:10.3390/s24196224)
Supplement: Supplementary file 1 [file sensors-24-06224-s001.zip › sensors-3149867-supplementary.pdf]

Supporting Information

Table S1. Primer set sequencing and their position in genomes.

	Primer Sequencing (5′–3′)	Primer position (*E. coli* O157:H7 EDL933 (AE005174))
F3-LAMP	CGGCGAACAGTAAGGAAG	2933714–2933731
B3-LAMP	ACTGGCCATGACTGGTAT	2933418–2933435
FIP-LAMP	TGGTGGTTCTGTTAGATCCAACAAGCATCG CGGAATATGG	(2933547–2933569) - (2933651–2933667)
BIP-LAMP	TTCGGCCAACGGCGATCATGAAACTCCTGG TTTATCTG	(2933503–2933520) - (2933457–2933476)
FLP-LAMP	CAAACCCTACACCATTATCTGT	(2933570–2933591)
BLP-LAMP	GCGGCGACATCATTATGGA	(2933480–2933498)

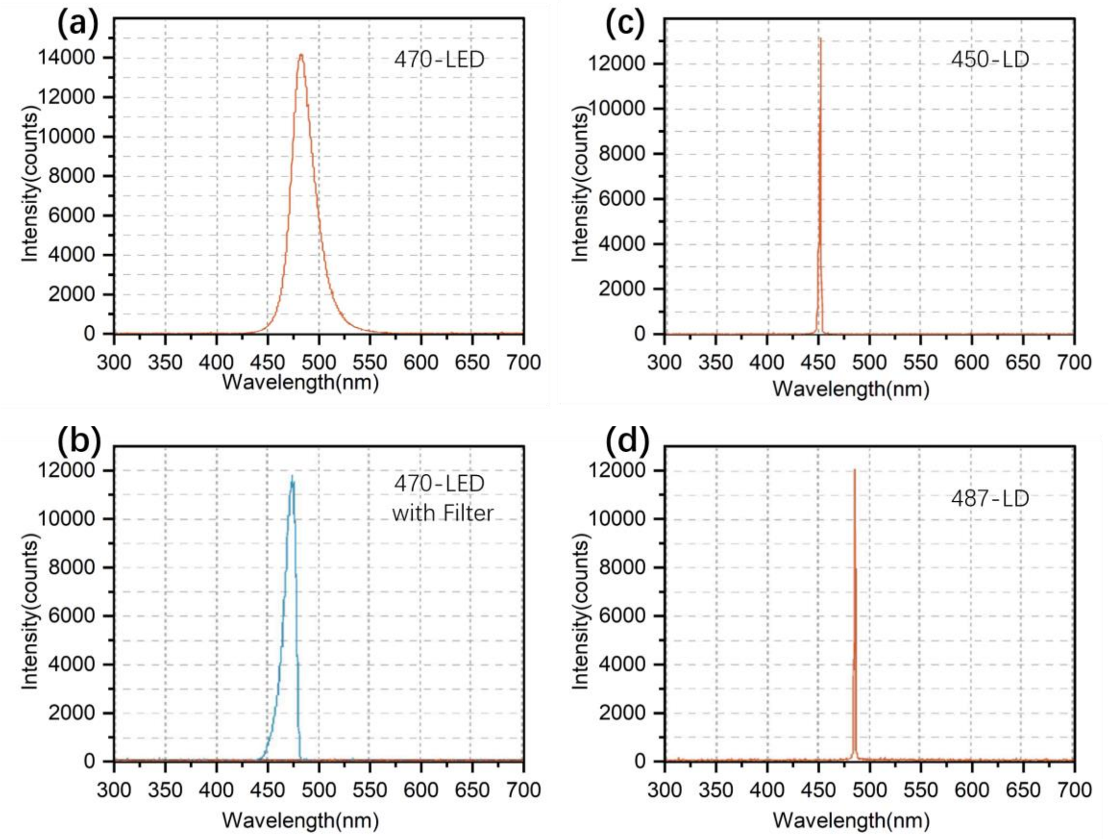

Figure S1. Spectrum of the light source. (a) The spectrum of a 470nm LED light source. (b) The spectrum of a 470nm LED light source with a filter attached. (c) The spectrum of a 450nm LD (Laser Diode) light source. (d) The spectrum of a 487nm LD (Laser Diode) light source.

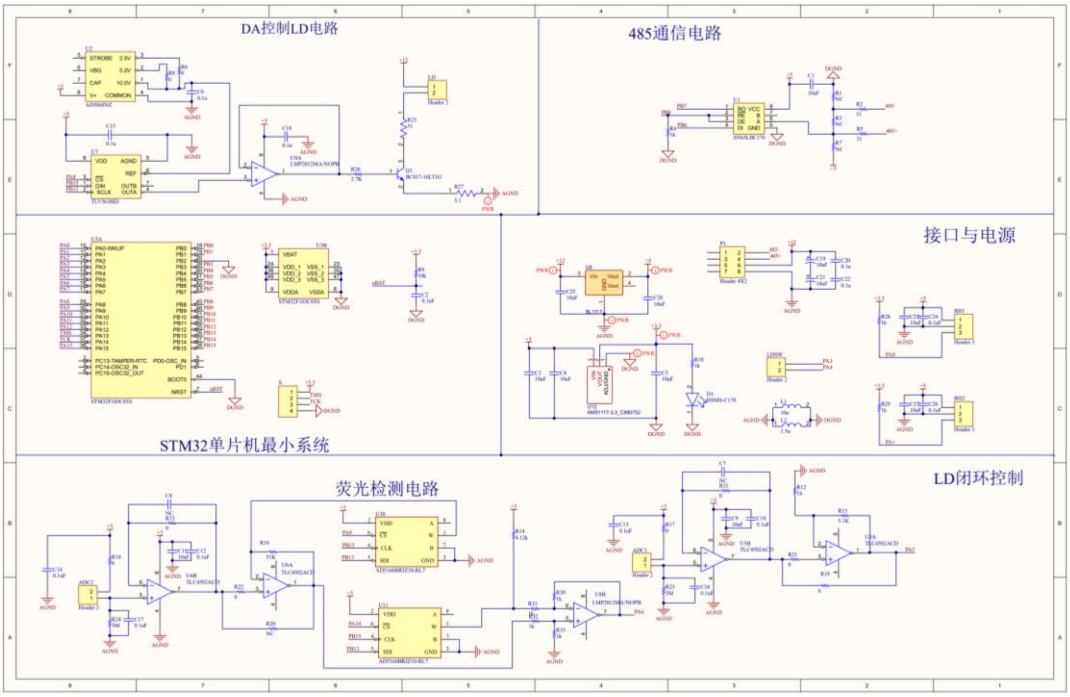

Figure S2. Circuit schematic of the control board.

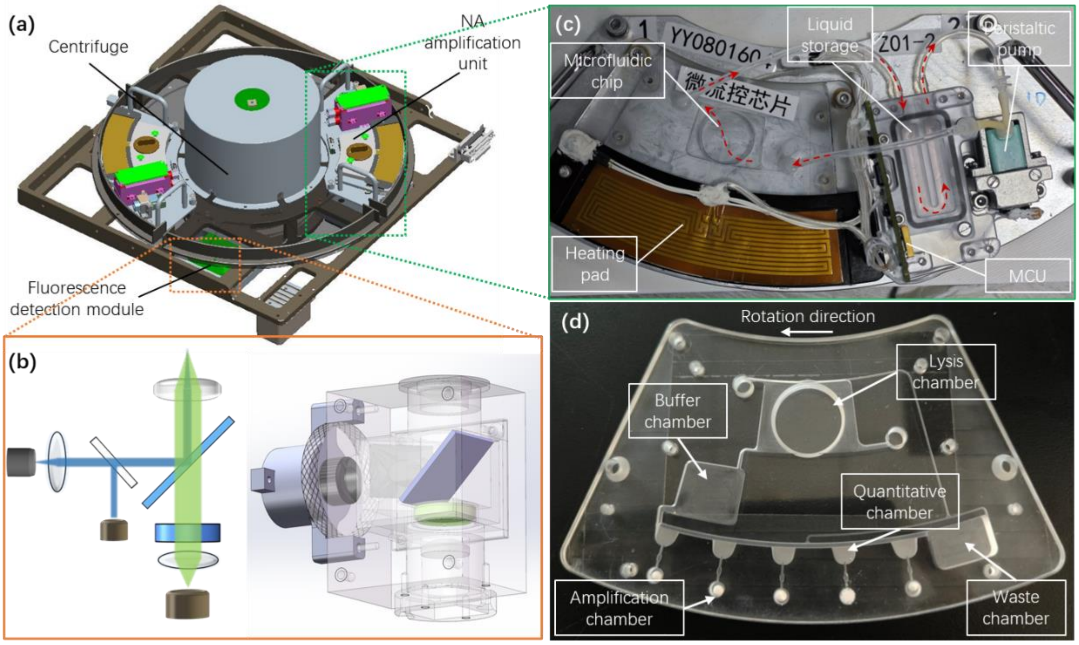

Figure S3. Configuration of the Nucleic Acid Amplification Detection System (a). The nucleic acid amplification unit is bolted onto the centrifuge, and the fluorescence detection module is mounted on the centrifuge frame. The centrifuge is hollowed out at the positions corresponding to the amplification chambers to ensure that the fluorescence signal is unobstructed. The fluorescence detection system is mounted on the bracket (b). The image of the nucleic acid amplification unit (c) and the centrifugal microfluidic chip (d).
